# Association between socioeconomic status and post-stroke functional outcome in deprived rural southern China: a population-based study

**DOI:** 10.1186/s12883-018-1017-4

**Published:** 2018-01-25

**Authors:** Fubing Ouyang, Ying Wang, Weixian Huang, Yicong Chen, Yuhui Zhao, Ge Dang, Chunbo Zhang, Yang Lin, Jinsheng Zeng

**Affiliations:** 1 Department of Neurology and Stroke Center, the First Affiliated Hospital of Sun Yat–Sen University, No.58 Zhongshan Road 2, Guangzhou, 510080 China; 2 Guangzhou Baiyunshan Qixing Pharmaceutical Co., Ltd, No.32 Yun Pu Road 1, Guangzhou, 510530 China

**Keywords:** Socioeconomic status, Epidemiology, Stroke recovery, Rural population, China

## Abstract

**Background:**

Data on the association between socioeconomic status and post-stroke functional outcome in developing countries is lacking. We aimed to evaluate the association in stroke survivors in deprived rural Southern China.

**Methods:**

We conducted door-to-door interviews and collected data using a structured questionnaire in stroke survivors from five fourth-class rural areas of Guangdong Province through a non-government initiated registry from August 2014 to March 2015. Descriptive statistics were used to provide information on the demographic, socioeconomic and clinical characteristics of the selected population. Univariate and multivariate logistic regression were used to examine the relationship of socioeconomic status indexed by self-reported average family income and functional impairment defined as a modified Rankin Scale of 3 to 5.

**Results:**

Among the 425 stroke survivors, 52.7% lived below the poverty line set by the local government. About 50% of patients suffered from functional impairment and required assistance in their daily life. Compared with their wealthier counterpart, stroke survivors with lower income were more likely to have functional impairment (OR 2.85, 95% CI 1.93—4.23). The effect size increased and remained significant after adjusting for possible confounding factors (OR 3.17, 95% CI 2.04—4.91).

**Conclusions:**

Poorer patients tend to have poorer post-stroke functional outcome. Primary and secondary strategies targeting underprivileged populations in less-developed areas are thus urgently needed in China.

**Electronic supplementary material:**

The online version of this article (10.1186/s12883-018-1017-4) contains supplementary material, which is available to authorized users.

## Background

The associations between socioeconomic status (SES), stroke incidence and mortality have long been recognized [1]. Mounting evidence suggests that people with a lower SES suffer from poorer functional outcome after stroke [2–6]. Many stroke survivors with severely impaired function are unable to return to work and rely on caregivers or healthcare services for a prolonged period of time, imposing a great burden on individual family and the whole society. Up-to-date evidence on the relationship between SES and post-stroke functional outcome has mainly come from studies conducted in high-income countries, limiting the generalization of findings to low- and middle-income countries.

China, the biggest middle-income country in the world, has approximately 2.5 million new stroke cases and 1.6 million total stroke-related deaths each year, and an estimated 7.5 million stroke survivors who suffer from long-term disability [7]. Emerging epidemiological data indicates that stroke burden has shifted from urban to rural areas [8–10]. Nonetheless, there is scarce data on patients living in deprived rural areas. Guangdong Province, located in Southern China and neighboring Hong Kong and Macao, has a much lower stroke incidence than Northern China and is thus often overlooked [7, 11]. Although it is one of the most prosperous parts of China, a large population resides in mountainous rural areas where there are with less-developed healthcare services. The difficult terrain, inconvenient transportation, communication problems, and lack of financial support from the government means that door-to-door data collection is rarely implemented [12].

In order to bridge this gap in the current knowledge, we interviewed stroke survivors in rural Southern China. The specific aims of this study were to compile data on the stroke burden and to examine the association between average family income and stroke functional outcome with adjustments for possible confounding factors.

## Methods

### Study population

**Fig. 1:**
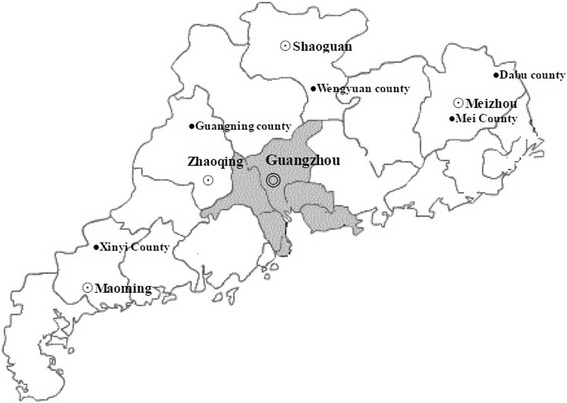
Distribution of the five fourth-class rural areas in Guangdong Province. The grey areas indicate the more developed areas located at the Pearl River Delta and adjacent to Hongkong and Macao [15]

The Baiyun Qixing Stroke Register was a non-government initiative that aimed to deliver medical assistance to patients with stroke from fourth-class rural areas with the worst economic level in Guangdong Province. It was launched in 2011 by Guangzhou Baiyunshan Qixing Pharmaceutical Company and the Red Cross Society of Guangdong Province and had documented 4612 patients so far. Between August 2014 and March 2015, we randomly selected patients from five fourth-class rural areas through this database (Fig. 1). Given that this simple registry recorded incomplete sociodemographic and clinical characteristics (including name, gender, age, address, phone number, family economic situation, stroke subtype, hospital admission), final case ascertainment was undertaken during face-to-face interviews. Inclusion criteria for patients were as follows: 18 years old and over, first or recurrent strokes diagnosed by local neurologists according to the World Health Organization criteria [13], having survived for more than 6 months post-stroke, and permanent residents of the rural areas. Patients who had died before the investigation, refused to participate were excluded. Ethical approval for the study was obtained from the Institutional Review Board of First Affiliated Hospital of Sun Yat-sen University.

### Data collection

Patients were interviewed by trained investigators from the Department of Neurology, the First Affiliated Hospital of Sun Yat-sen University using a structured questionnaire. The study staff also was composed of a Red Cross member, a village committee cadres and a village physician who provides primary care services in local areas. Good clinical practice guidelines in accordance with the Declaration of Helsinki were used. Questions were answered by patients or their proxies in case of verbal/cognitive impairment or severe illness. Written informed consent was obtained. Information was retrieved on sociodemographic and clinical characteristics. Blood pressures were measured three times at both brachial arteries using standard mercury sphygmomanometers after the patient had rested for at least 5 min in a seated position. Blood samples were not drawn for test due to a lack of funding and equipment.

### Interpretation of data

Self-reported monthly family income per capita was used as a single indicator of SES which was more applicable than education attainment and occupation in the present study due to the fact that the majority of rural residents have achieved relatively low level of education and are farmers [1, 14]. The total monthly income included salaries, pensions, and earnings from homegrown products (rice, vegetables). We further divided patients into lower- and higher-income groups according to the latest poverty line (260 Chinese Yuan Renminbi, RMB, per capita per month, set by the Government of Guangdong), equivalent to around US $ 41 based on an exchange rate of US $1 to RMB6.4 [15].

Presence of hypertension, diabetes mellitus, hyperlipidemia and atrial fibrillation were determined according to self-reports of disease history or of current treatment. Hypertension control was defined according to the Eighth Joint National Committee on the Prevention, Detection, Evaluation and Treatment of Hypertension (JNC-8) [16]. Blood Glucose control was defined as the latest self-monitoring fasting blood glucose < 7.0 mmol/L [17]. Current smoking was defined as more than 1 cigarette/day for at least 1 year. Current alcohol consumption was defined as more than 14 g of pure alcohol/day for at least 1 year [10]. Hospitals were classified as level 1 (community hospitals with only basic medical facilities and very limited inpatient capacity), level 2 (hospitals with at least 100 inpatient beds providing acute medical care and preventative care services to populations of at least 100,000), and level 3 (major tertiary referral centers in provincial capitals and major cities) [18]. Functional outcome was assessed using the modified Rankin Scale (mRS) score. Based on the scores, patients were divided into two groups: functional independence (mRS 0–2) and functional impairment (mRS 3–5) [19].

### Statistical analysis

Descriptive statistics were used to assess patients’ sociodemographic and clinical characteristics. Unpaired t-test, χ^2^ test, Fisher’s exact test, and Mann–Whitney U tests were used to perform univariate comparisons. To examine the association between average monthly family income and functional outcome, we used univariate logistic regression to calculate the crude odd ratio (OR) and its 95% confidential interval (CI), and multivariate logistic regression with stepwise method to adjust possible confounding factors. First, we added age and sex to the model. Second, we added other possible confounders that were of statistical significance in univariate comparison. The data were analyzed via SPSS version 20.0 (SPSS Inc., Chicago, IL, USA).

## Results

### Study population

A total of 477 patients were identified through the database. Of these, 42 patients died and 10 had a stroke attack within the previous 6 months. This left a total of 425 eligible stroke survivors (a response rate of 89.1%) who had clinically suspected or neuroimaging-confirmed stroke.

### Patient characteristics

Basic characteristics of patients are presented in Table 1. The 425 patients had a male to female ratio of approximately 1.76:1. The mean ± SD age of first stroke occurrence was younger in males than in females (58.0 ± 11.5 versus 61.7 ± 10.7 years, *P* = 0.001). The median monthly family income per capital was RMB250/$39 (IQR RMB83/$13-RMB439/$69). A total of 52.7% of households lived below the poverty line. All patients were insured by the New Rural Cooperative Medical System (NRCMS) financed largely from the government [20].

**Table 1:** Characteristics and functional outcome of the sample participants according to income level

Variables	Total no./total (%)	Family average monthly income ≤ RMB 260 no./total (%)	Family average monthly income > RMB260 no./total (%)	*P*
Sociodemographic characteristics				
Male gender	271/425(63.8)	146/224(65.2)	125/201(62.2)	0.52
Age of occurrence,years	60.7 ± 11.4	61.2 ± 11.1	60.1 ± 11.7	0.33^a^
< 65	265/415(63.9)	136/218(62.4)	129/197(65.5)	0.51
Marital status				
Married	319/425(75.1)	166/224((74.1)	153/201((76.1)	0.63
Unmarried/devorced/widowed	106/425(24.9)	58/224(25.9)	48/201(23.9)	
Living alone	25/425(5.9)	16/224(7.1)	9/201(4.5)	0.24
Unemployed/retired	303/425(71.3)	162/224(72.3)	141/201(70.1)	0.62
Comorbidities and risk factors				
Hypertension	341/425(80.2)	181/224(80.8)	160/201(79.6)	0.76
Use of anti-hypertensive drugs	245/341(71.8)	132/181(72.9)	113/160(70.6)	0.64
Control of hypertension	55/296(18.6)	25/149(16.8)	30/147(20.4)	0.42
Diabetes mellitus	71/425(16.7)	34/224(15.2)	37/201(18.4)	0.37
Use of hypoglycemic drugs	50/71(70.4)	23/34(67.6)	27/37(73.0)	0.62
Control of fasting blood glucose	7/71(9.9)	5/34(27.8)	2/37(7.7)	0.17
Hyperlipidaemia	88/425(20.7)	45/224(20.1)	43/201(21.4)	0.74
Use of lipid-lowering drugs	33/88(37.5)	13/45(28.9)	20/43(46.5)	0.09
Artrial fibrillation	12/425(2.8)	7/224(3.1)	5/201(2.5)	0.69
Use of anticoagulant drugs	0/12	0/7	0/5	
Coronary heart diseases	39/425(9.2)	19/224(8.5)	20/201(10.0)	0.60
No comorbidities	79/425(18.6)	39(17.4)	40(19.9)	0.51
Family history	60/425(14.1)	27/224(12.1)	33/201(16.4)	0.20
Current smoking	67/425(15.8)	31/224(13.8)	36/201(17.9)	0.25
Current alcohol consumption	11/425(2.6)	5/224(2.2)	6/201(3.0)	0.63
Stroke care services				
Stroke type				
Ischemic stroke	291/425(68.5)	139/224(62.0)	152/201(75.6)	0.001
Hemorrhagic stroke	90/425(21.2)	51/224(22.8)	39/201(19.4)	
Unknown	44/425(10.4)	34/224(15.2)	10/201(5.0)	
Neuroimaging diagnosis				
CT brain scan	380/425(89.4)	190/224(84.8)	190/201(94.5)	0.001
MRI brain scan	142/425(33.4)	63/224(28.3)	79/201(39.3)	0.02
None	44/425(10.4)	34/224(15.2)	10/201(5.0)	0.001
Course of stroke,years	4.0(2.0,6.0)	4.0(2.0,6.0)	4.0(2.0,6.0)	0.99^c^
Previous stroke				
0	291/425(68.5)	146/224(65.2)	145/201(72.1)	0.28
1	89/425(20.9)	53/224(23.7)	36/201(17.9)	
≥ 2	45/425(10.6)	25/224(11.2)	20/201(10.0)	
Awareness of stroke warning sign^d^	196/425(46.1)	104/224(46.4)	92/201(45.8)	0.89
Time from onset to emergency room (hours)				
≤ 2	205/409(50.1)	107/215(49.8)	98/194(50.5)	0.71
2–6	103/409(25.2)	53/215(24.7)	50/194(25.8)	
6–24	36/409(8.8)	17/215(7.9)	19/194(9.8)	
> 24	65/409(15.9)	38/215(17.7)	27/194(13.9)	
Use of emergency ambulance	98/425(23.1)	58/224(25.0)	42/201(20.9)	0.32
Level of hospital admitted				
1	105/415(25.3)	54/218(24.8)	51/197(25.9)	0.04
2	250/415(60.2)	141/218(64.7)	109/197(55.3)	
3	60/415(14.5)	23/218(10.6)	37/197(18.8)	
Ischemic stroke				
Intravenous thrombolysis	6/291(2.1)	1/139(0.7)	5/152(3.3)	0.26^b^
Antiplatelet therapy after discharge(Aspirin)	107/291(36.8)	51/139(36.7)	56/152(36.8)	0.98
Antiplatelet Therapy after discharge(Clopidogrel)	7/291(2.4)	2/139(1.4)	5/152(3.3)	0.52
Hemorrhagic stroke				
Surgery	26/90(28.9)	14/51(27.5)	12/39(30.8)	0.73
Rehabilitation	187/425(44.0)	99/224(44.2)	88/201(43.8)	0.93
Duration of rehabilitation(weeks)				
< 4	94/174(54.0)	45/89(50.6)	49/85(57.6)	0.54^c^
4–8	24/174(13.8)	15/89(16.9)	9/85(10.6)	
8–12	5/174(2.9)	3/89(3.4)	2/85(2.4)	
> 12	51/174(29.3)	26/89(29.2)	25/85(29.4)	
Inhospitalization in previous year	106/425(24.9)	50/224(22.3)	56/201(27.9)	0.19
Outpatient attendance in previous month				
Level 1 hospital	88/425(20.7)	41/224(18.3)	47/201(23.4)	0.20
Level 2–3 hospital	30/425(7.1)	9/224(4.0)	21/201(10.4)	0.01
Modified Rankin Scale Score				
0–2	209/425(49.1)	83/224(37.1)	126/201(62.7)	< 0.001
3–5	216/425(50.9)	141/224(62.9)	75/201(37.3)	

*CT* indicates Computed Tomography, *MRI* indicates Magnetic Resonance Imaging, *no.* indicates number, *RMB* indicates Chinese Yuan Renminbi

*P* values are based on χ^2^ test if not indicated otherwise

^a^*P* value based on unpaired *t* test

^b^*P* value based on Fisher’s exact test

^c^*P* value based on Mann–Whitney *U* test

^d^Stroke warning signs include the sudden loss of motor or sensory function, speech difficulty, changes in vision, gait, severe headache, vertigo/dizziness or a depressed level of consciousness

### Comorbidities and risk factors

We calculated the frequency distribution of hypertension (80.2%), hyperlipidemia (20.7%), diabetes mellitus (16.7%), coronary heart diseases (9.2%), atrial fibrillation (2.8%) and current smoking (15.8%). The probability of current smoking was higher in males than females (23.6% versus 1.9%, *P* < 0.001), while the proportion of diabetes mellitus was marginally higher in females than males (21.4% versus 14.0%, *P* = 0.049).

### Stroke care delivery in acute and chronic phase

**Fig. 2:**
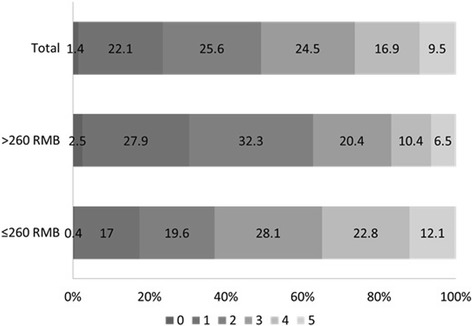
Distribution of post-stroke functional outcome in total and in groups stratified by income level

Patients had survived for a median (IQR) of 4 (2–6) years after stroke at the time of the study. Up to 68.5% of strokes were attributed to ischemic stroke, 20.7% to primary intracerebral hemorrhage and 1.4% to subarachnoid hemorrhage. Those who were treated in a level 2–3 hospital, who had the stroke attack within the past 10 years, and who had higher income were more likely to have received neuroimaging diagnosis (all *P* ≤ 0.001). With regards to healthcare for chronic phase post-stroke, 44.0% of patients received rehabilitation therapy. In all, half of the stroke survivors lived with functional impairment (Fig. 2).

### Income and functional outcome post-stroke

Patient characteristics and functional outcome according to income level were also presented in Table 1. Compared with patients with higher income, lower-income patients were less likely to have attended a level 3 hospital (10.6% versus 18.8%, *P* = 0.01, with level 2 hospital as reference), and have received a neuroimaging (CT or MRI)-based diagnosis (84.8% versus 95.0%, *P* = 0.001). However, in neuroimaging-confirmed cases, stroke type did not differ between the two groups. In chronic phase, lower-income patients were less likely to have attended outpatient services in level 2–3 hospitals (4.0% versus 10.4%, *P* = 0.01). There were no significant differences in terms of sex, age, occupational status, and comorbidities.

Univariate logistic regression revealed that the lower-income group was more likely to experience poor functional outcome compared with the higher-income group (OR 2.85, 95%CI 1.93–4.23, *P* < 0.001). This effect slightly increased after adjustment by age and sex (OR 2.98, 95%CI 1.987–4.477, *P* < 0.001). Poor functional outcome was also positively associated with older age at onset, not living alone, being retired or unemployed, being a non-current smoker, hemorrhagic stroke, recurrent stroke, call for emergency services and rehabilitation (all *P* < 0.05, see Additional file 1). After adjusting for all these potential confounding variables, the effect size remained statistically significant and was even larger (OR 3.17, 95% CI 2.04–4.91, *P* < 0.001) (Table 2).

**Table 2:** The association between income and functional outcome in stroke survivors in five rural areas

	OR	95%CI	*P*
Unadjusted			
Average monthly income of the family ≤ 260 RMB	2.85	(1.93, 4.23)	< 0.001
Adjusted for age and sex			
Average monthly income of the family ≤ 260 RMB	2.98	(1.99, 4.48)	< 0.001
Age of occurrence ≥ 65 years	1.48	(1.10, 1.98)	0.009
Male	0.70	(0.46, 1.07)	0.10
Adjusted for all possible confounders			
Average monthly income of the family ≤ 260 RMB	3.17	(2.04, 4.91)	< 0.001
Age of occurrence ≥ 65 years	1.42	(1.03, 1.97)	0.03
Male	0.97	(0.60, 1.55)	0.88
Not living alonoe	9.76	(2.69, 35.40)	0.001
Currently unemployed/retired	1.80	(1.10, 2.95)	0.01
Currently smoking	0.42	(0.21, 0.82)	0.01
History of previous stroke	1.83	(1.14, 2.95)	0.01
Use of emergency service system	2.20	(1.29, 3.73)	0.004
Rehabilitation	1.80	(1.14, 2.82)	0.011

*OR* indicates odd ratio, *CI* indicates confidential interval

## Discussion

Limited information exists on the stroke epidemiology in less-developed rural China, and few investigations examined the association between SES and long-term disability in stroke survivors. In this population-based cross-sectional study, we took a snapshot of the heavy burden of stroke rural Southern China. This area has prevalent yet insufficiently controlled vascular risk factors, inadequate stroke care delivery and a high proportion of disabled stroke survivors. Furthermore, we found that patients with lower SES, indexed by average monthly income of the family, were at greater risk of long-term functional impairment, even after adjusting for possible demographic and clinical characteristics.

Guangdong has enjoyed the fruits of rapid economic development over the past three decades,with its overall Gross Domestic Product ranking first in Mainland China, which has brought with it life-style changes (i.e., increased consumption of fatty and energy-dense foods, and lack of physical activity). Such factors may lead to a heavier stroke burden, particularly in less-developed rural areas with inadequate healthcare resources and suboptimal implementation of evidenced-based practice [8, 9, 21]. Our data support these growing concerns.

In line with other epidemiological studies conducted in China, the profile of comorbidities and stroke risk factors in our study was similar to those of developed countries [9, 22, 23]. Among them hypertension remained the most significant one for all types of stroke [7]. Although 71.8% of patients with hypertension were taking antihypertensive medication, only 18.6% showed evidence of adequate blood pressure control. Indeed, the poor treatment and control of hypertension in Northern China’s rural population has previously been reported [24]. The rate of fasting blood glucose monitor and control was also low. Of the 291 ischemic stroke patients discharged from hospital, 60.8% did not comply with antiplatelet therapy. These findings suggest that long-term compliance with secondary stroke prevention in socioeconomically disadvantaged population is still poor [12], including incorrect discontinuation, doses reduction and a preference to Chinese traditional herb medicine (47.8%).

A large proportion of patients had undergone CT scanning. The use of CT scanning in rural areas of China has been previously reported as “low” compared with urban areas but precise data is lacking [11]. CT scanning is now widely available in almost all county-level and city-level hospitals, resulting in improvement of stroke diagnosis and classification. Consistent with previous studies conducted in China, ischemic stroke was found to be the most common type. However, we could not obtain the time interval from stroke onset or admission to imaging, which is a more critical aspect in the evaluation of acute stroke management [25].

In terms of emergency transport, less than one fourth of patients called for an emergency ambulance after symptom onset. Of all ischemic strokes, very few received intravenous thrombolysis in time. Unawareness of stroke warning signs, pre-hospital delay owing to long transportation as the majority of patients lived in remote areas, and in-hospital delay such as prolonged time from admission to imaging, were some major explanations that might be offered for this low rate of thrombolytic therapy. Moreover, all 6 cases who had received intravenous thrombolysis were administered with urokinase but not tPA, probably as tPA is expensive [11, 12]. These data suggest that the emergency service system for acute stroke are not well established in these rural areas [12, 26]. Additionally, few patients continued their rehabilitation programs after being discharged. Since the types, duration and intensity of rehabilitation were not measured, the association between rehabilitation and functional outcome was inclusive.

In terms of the role of health care system, all patients were insured by NRCMS in our study [20]. NRCMS is a wide health care system launched by the Chinese government in 2003, aiming to improve the rural residents’ access to health services, and reduce their risk of catastrophic medical payments resulting from major diseases [27, 28]. It is financed by combined contributions from central and local government, and individual rural households [27, 28]. The government’s subsidy financing level increased gradually and reached RMB 340 by 2013, accounting for more than three quarters of the pooling funds [29, 30]. However, recent studies suggested that NRCMS did not sufficiently alleviate the financial burden of rural residents at the current funding level, as the reimbursement rate are relatively low [20, 31, 32]. NRCMS reimburses approximately 30% of inpatient expenditures in practice [20]. Unfortunately, we failed to collect individual data on the out-of-pocket and NRCMS-reimbursed expenses in the present study. Wang et al. [20] found that out-of-pocket payments for treatment of stroke averaged RMB3028.4 per capita in 2008 in poor rural areas of China. Hence, we can infer that, in the poorest group with monthly income of no more than RMB 260 per capita in our study, medical expenditures still pose a huge financial burden on the households. We found that the destitute population was less likely to undertake neuroimaging for stroke diagnosis compared with their wealthier counterparts. One possible explanation is that poorer rural residents may cut down their medical expenses to avoid catastrophic out-of-pocket payments [20]. Additionally, the low-income group prefers to attend low-cost village clinics and township-level hospitals where CT or MRI imaging equipment is still lacking [11].

The finding that low SES was associated with a greater risk of post-stroke functional impairment is in line with results of several hospital-based prospective cohort studies [2–4]. However, all of these studies were hospital-based and used different indicators of SES. Data from population-based studies, particularly conducted in developing counties remains scarce.

It has been suggested that the socioeconomic disparity in stroke outcome cannot be fully explained by variations in patients’ clinical and demographic characteristics [1–5]. Similarly, the association we observed could not be explained by the differential distribution of risk factors or unequal access to healthcare between the two income groups. There are likely several explanations for our findings. First, we did not draw blood samples to test blood glucose and lipids, nor did we perform electrocardiogram to detect any arrhythmia. Thus our assessment of comorbidities and risk factors were based on self-report results, which may result in an underestimation of their prevalence compared with their biochemical gold standard [33]. Second, the association may have been driven by factors that were not measured in the present study, such as baseline stroke severity measured by the National Institutes of Health Stroke Scale. It has been suggested that the impoverished population tend to suffer from more severe stroke [34–36]; for another, patients with milder stroke in the lower income group would be less likely to seek medical help due to medical costs, which may lead to imbalance of stroke severity in the two groups. Third, we did not collect information on the timing of specific care processes, which may reflect the quality of care and could thus partly explain some of the association between SES and functional outcome. Nor did we collect data on other comorbidities that might have a negative impact on stroke prognosis, such as chronic lung, liver or renal diseases [37, 38].

Our study suffered from some other limitations. As this study was cross-sectional and had a relatively small sample, we failed to uncover any cause-and-effect relationship between SES and functional outcome after stroke. Part of the association we observed may be due to the impact of stroke on average family income, as catastrophic payment for stroke treatment and loss of labor force may in turn push the household below the absolute poverty line. The association may also be influenced by the “survivor effect” (those who died may have had more severe disability), and “recall bias” (e.g. in those with a long course of disease or at very old age) [13]. In addition, there were problems when average monthly income of the family was used as a single indicator of SES. Compared with educational attainment and occupation status, income is not that stable and may vary over time [1]. Furthermore, patients in rural areas may underestimate their earnings and the value of their home-grown products [13].

Evidently, conducting of ideal population-based epidemiological stroke studies is challenging for us. As there are no well-established systems in local communities to keep medical records, or to monitor and follow up patients, a door-to-door interview is the realistic choice for us, though it was manpower, time and material resources-consuming [7, 11]. Nonetheless, we feel that future studies should also attempt to overcome these obstacles in order to enhance our understanding of SES and stroke outcome. Furthermore, primary and secondary stroke care strategies targeting underprivileged groups should be developed. It is also necessary to improve the effectiveness of NRCMS by improving the reimbursement rate, increasing subsidizes, targeting the poorest households, and providing them with additional medical aids or financial assistance [20, 31, 32].

## Conclusions

This study provides some rough ideas of the stroke burden in Southern China, and adds to the accumulative evidence that patients with a low SES are not only more likely to have a stroke attack but also more likely to be disabled after stroke. This has important implications for public health policy makers. Primary and secondary stroke care strategies targeting underprivileged groups should be developed and healthcare insurance systems should be improved to ensure that all patients have equal access to diagnostic, treatment and rehabilitation services regardless of their SES. Epidemiological studies with high methodological quality, especially with prospective design should be conducted to further investigate the relationship between SES and stroke outcomes, not only in rural China but also in poverty-stricken areas worldwide [7, 11].

## Additional file

Additional file 1: Characteristics of the sample participants according to functional outcome. (DOCX 24 kb)

## Abbreviations

CI: Confidential interval
CT: Computed tomography
IQR: Interquartile range
JNC: Joint National Committee
MRI: Magnetic resonance imaging
mRS: Modified Rankin Scale
NRCMS: New Rural Cooperative Medical System
OR: Odd ratio
RMB: Chinese Yuan Renminbi
SD: Standard deviation
SES: Socioeconomic status
US: United States

## References

[1] Marshall IJ, Wang Y, Crichton S, McKevitt C, Rudd AG, Wolfe CD (2015). The effects of socioeconomic status on stroke risk and outcomes. Lancet Neurol.

[2] van den Bos GA, Smits JP, Westert GP, van Straten A (2002). Socioeconomic variations in the course of stroke: unequal health outcomes, equal care?. J Epidemiol Community Health.

[3] Grube MM, Koennecke HC, Walter G, Thümmler J, Meisel A, Wellwood I, Heuschmann PU (2012). Association between socioeconomic status and functional impairment 3 months after ischemic stroke: the Berlin stroke register. Stroke.

[4] Bettger JP, Zhao X, Bushnell C, Zimmer L, Pan W, Williams LS, Peterson ED (2014). The association between socioeconomic status and disability after stroke: findings from the adherence eValuation after ischemic stroke longitudinal (AVAIL) registry. BMC Public Health.

[5] Chen R, Crichton S, McKevitt C, Rudd AG, Sheldenkar A, Wolfe CD (2015). Association between socioeconomic deprivation and functional impairment after stroke: the South London stroke register. Stroke.

[6] Sturm JW, Donnan GA, Dewey HM, Macdonell RA, Gilligan AK, Thrift AG (2004). Determinants of handicap after stroke: the north East Melbourne stroke incidence study (NEMESIS). Stroke.

[7] Liu L, Wang D, Wong KS, Wang Y (2011). Stroke and stroke care in China: huge burden, significant workload, and a national priority. Stroke.

[8] Zhang XH, Guan T, Mao J, Liu L (2007). Disparity and its time trends in stroke mortality between urban and rural populations in China 1987 to 2001: changing patterns and their implications for public health policy. Stroke.

[9] Wang J, An Z, Li B, Yang LI, Tu J, Gu H, Zhan C, Liu B, Su TC, Ning X (2015). Increasing stroke incidence and prevalence of risk factors in a low-income Chinese population. Neurology.

[10] Tang X, Laskowitz DT, He L, Østbye T, Bettger JP, Cao Y, Li N, Li J, Zhang Z, Liu J, Yu L, Xu H, Hu Y, Goldstein LB (2015). Neighborhood socioeconomic status and the prevalence of stroke and coronary heart disease in rural China: a population-based study. Int J Stroke.

[11] Liu M, Wu B, Wang WZ, Lee LM, Zhang SH, Kong LZ (2007). Stroke in China: epidemiology, prevention, and management strategies. Lancet Neurol.

[12] Joubert J, Prentice LF, Moulin T, Liaw ST, Joubert LB, Preux PM, Ware D, Medeiros de Bustos E, McLean A (2008). Stroke in rural areas and small communities. Stroke.

[13] Hatano S (1976). Experience from a multicentre stroke register: a preliminary report. Bull World Health Organ.

[14] Xu F, Ah Tse L, Yin X, Yu IT, Griffiths S (2008). Impact of socio-economic factors on stroke prevalence among urban and rural residents in mainland China. BMC Public Health.

[15] Urban and rural subsistence allowance system. Department of Civil Affairs of Guangdong Province.http://www.gdmz.gov.cn/gdmz/uploads/zcfg/shbz/201503/P020150324594766665942.pdf. Accessed 24 Jan 2018.

[16] James PA, Oparil S, Carter BL, Cushman WC, Dennison-Himmelfarb C, Handler J, Lackland DT, ML LF, TD MK, Ogedegbe O, Smith SC, Svetkey LP, Taler SJ, Townsend RR, Wright JT, Narva AS, Ortiz E (2014). 2014 evidence-based guideline for the management of high blood pressure in adults: report from the panel members appointed to the eighth joint National Committee (JNC 8). JAMA.

[17] Alberti KG, Zimmet PZ (1998). Definition, diagnosis and classification of diabetes mellitus and its complications. Part 1: diagnosis and classification of diabetes mellitus provisional report of a WHO consultation. Diabet Med.

[18] Wei JW, Wang JG, Huang Y, Liu M, Wu Y, Wong LK, Cheng Y, Xu E, Yang Q, Arima H, Heeley EL, Anderson CS, ChinaQUEST Investigators (2010). Secondary prevention of ischemic stroke in urban China. Stroke.

[19] van Swieten JC, Koudstaal PJ, Visser MC, Schouten HJ, van Gijn J (1988). Interobserver agreement for the assessment of handicap in stroke patients. Stroke.

[20] Wang Q, Liu H, Lu ZX, Luo Q, Liu JA (2014). Role of the new rural cooperative medical system in alleviating catastrophic medical payments for hypertension, stroke and coronary heart disease in poor rural areas of China. BMC Public Health.

[21] Sposato LA (2015). Worsening risk factors and more strokes: the dark side of economic growth?. Neurology.

[22] He L, Tang X, Song Y, Li N, Li J, Zhang Z, Liu J, Yu L, Xu H, Zhang J, Hu Y (2012). Prevalence of cardiovascular disease and risk factors in a rural district of Beijing, China: a population-based survey of 58,308 residents. BMC Public Health.

[23] Jia Q, Liu L, Wang Y (2011). Risk factors and prevention of stroke in the Chinese population. J Stroke Cerebrovasc Dis.

[24] Wang J, Ning X, Yang L, Lu H, Tu J, Jin W, Zhang W, Su TC (2014). Trends of hypertension prevalence, awareness, treatment and control in rural areas of northern China during 1991-2011. J Hum Hypertens.

[25] Langagergaard V, Palnum KH, Mehnert F, Ingeman A, Krogh BR, Bartels P, Johnsen SP (2011). Socioeconomic differences in quality of care and clinical outcome after stroke: a nationwide population-based study. Stroke.

[26] Jia Q, Liu LP, Wang YJ (2010). Stroke in China. Clin Exp Pharmacol Physiol.

[27] Li C, Hou Y, Sun M, Lu J, Wang Y, Li X, Chang F, Hao M (2015). An evaluation of China's new rural cooperative medical system: achievements and inadequacies from policy goals. BMC Public Health.

[28] Liang X, Guo H, Jin C, Peng X, Zhang X (2012). The effect of new cooperative medical scheme on health outcomes and alleviating catastrophic health expenditure in China: a systematic review. PLoS One.

[29] Zhu K, Zhang L, Yuan S, Zhang X, Zhang Z (2017). Health financing and integration of urban and rural residents' basic medical insurance systems in China. Int J Equity Health.

[30] Zou J, Yang W, Cook DM, Yuan Z, Zhang L, Wang X (2016). New cooperative medical financing policy and hospitalization in rural China: multi-stage cross-sectional surveys. Int Health.

[31] Guo N, Iversen T, Lu M, Wang J, Shi L (2016). Does the new cooperative medical scheme reduce inequality in catastrophic health expenditure in rural China?. BMC Health Serv Res.

[32] Heeley E, Anderson CS, Huang Y, Jan S, Li Y, Liu M, Sun J, Xu E, Wu Y, Yang Q, Zhang J, Zhang S, Wang J, ChinaQUEST Investigators (2009). Role of health insurance in averting economic hardship in families after acute stroke in China. Stroke.

[33] Kanjilal S, Gregg EW, Cheng YJ, Zhang P, Nelson DE, Mensah G, Beckles GL (2006). Socioeconomic status and trends in disparities in 4 major risk factors for cardiovascular disease among US adults, 1971-2002. Arch Intern Med.

[34] Rey V, Faouzi M, Huchmand-Zadeh M, Michel P (2011). Stroke initial severity and outcome relative to insurance status in a universal health care system in Switzerland. Eur J Neurol.

[35] Aslanyan S, Weir CJ, Lees KR, Reid JL, McInnes GT (2003). Effect of area-based deprivation on the severity, subtype, and outcome of ischemic stroke. Stroke.

[36] Kleindorfer D, Lindsell C, Alwell KA, Moomaw CJ, Woo D, Flaherty ML (2012). Patients living in impoverished areas have more severe ischemic strokes. Stroke.

[37] Cesaroni G, Agabiti N, Forastiere F, Perucci CA (2009). Socioeconomic differences in stroke incidence and prognosis under a universal healthcare system. Stroke.

[38] Weir NU, Gunkel A, McDowall M, Dennis MS (2005). Study of the relationship between social deprivation and outcome after stroke. Stroke.

