# Supplementary material for: Association between socioeconomic status and post-stroke functional outcome in deprived rural southern China: a population-based study
Source: BMC Neurol. 2018 Jan 25;18:12. doi: 10.1186/s12883-018-1017-4 (PMC5785852; doi:10.1186/s12883-018-1017-4)
Supplement: Additional file 1: — Characteristics of the sample participants according to functional outcome. (DOCX 24 kb) [file 12883_2018_1017_MOESM1_ESM.docx]

**Additional Table . Characteristics of the sample participants according to functional outcome.**

| **Variables** | | **mRS≤2** | **mRS≥3** | ***P* value** |
| --- | --- | --- | --- | --- |
|  |  | **no./total(%)** | **no./total(%)** |  |
| **Sociodemographic characteristics** | |  |  |  |
| Male gender | | 140/209(67.0) | 131/216(60.6) | 0.17 |
| Age of occurrence (years) | | 58.99±10.67 | 62.35±11.88 | 0.003^a^ |
|  | <65 | 146/204(71.6) | 119/211(56.4) | 0.001 |
|  | ≥65 | 58/204(28.4) | 92/211(43.6) |  |
| Marital status | |  |  |  |
|  | Married | 155/209(74.2) | 164/216(75.9) | 0.67 |
|  | Unmarried/devorced/widowed | 54/209(25.8) | 52/216(24.1) |  |
| Living alone | | 22/209(10.5) | 3/216(1.4) | <0.001 |
| Retired/unemployed | | 138/209(66.0) | 165/209(76.4) | 0.02 |
| Average monthly income of the family | | 333.3(158.6, 500) | 150.0(50.0, 349.2) | <0.001 |
|  | ≤260 | 83/209(39.7) | 141/216(65.3) | <0.001 |
|  | >260 | 126/209(60.3) | 75/216(34.7) |  |
| **Comorbidities and risk factors** | |  |  |  |
| Hypertension | | 165/209(78.9) | 176/216(81.5) | 0.51 |
|  | Use of anti-hypertensive drugs | 113/165(68.5) | 132/176(75.0) | 0.18 |
|  | Control of hypertension | 23/149(15.4) | 32/147(21.8) | 0.16 |
| Diabetes mellitus | | 37/209(17.7) | 34/216(15.7) | 0.59 |
|  | Use of hypoglycemic drugs | 25/37(67.6) | 25/34(73.5) | 0.58 |
|  | Control of fasting blood glucose | 4/24(16.7) | 3/20(15.0) | 1.00 |
| Hyperlipidaemia | | 42/209(20.1) | 46/216(21.3) | 0.76 |
|  | Use of lipid-lowering drugs | 18/42(42.9) | 15/46(32.6) | 0.32 |
| Artrial fibrillation | | 5/209(2.4) | 7/216(3.2) | 0.60 |
|  | Use of anticoagulant drugs | 0/5 | 0/7 |  |
| Coronary heart diseases | | 25/209(12.0) | 14/216(6.5) | 0.05 |
| Family history | | 34/209(16.3) | 26/216(12.0) | 0.21 |
| Current smoking | | 48/209(23.0) | 19/216(8.8) | <0.001 |
| Current alcohol consumption | | 9/209(4.3) | 2/216(0.9) | 0.06 |
| **Stroke care delivery** | |  |  |  |
| Stroke type(confirmed by neuroimaging) | |  |  |  |
|  | Ischemic stroke | 151/185(81.6) | 140/196(71.4) | 0.02 |
|  | Hemorrhagic stroke | 34/185(18.4) | 56/196(28.6) |  |
| Neuroimaging diagnosis | |  |  |  |
|  | CT brain scan | 184/209(88.0) | 196/216(90.7) | 0.37 |
|  | MRI brain scan | 66/209(31.6) | 76/216(35.2) | 0.43 |
|  | None | 24/209(11.5) | 20/216(9.3) | 0.45 |
| Course of stroke(years) | | 4.0(2.0, 6.0) | 4(2.0, 7.0) | 0.97^c^ |
| Previous stroke | |  |  |  |
|  | 0 | 161(77.0) | 130(60.2) | <0.001^d^ |
|  | 1 | 35(16.7) | 54(25.0) |  |
|  | ≥2 | 13(6.2) | 32(14.8) |  |

**Additional Table (continued)**

| **Variables** | | **mRS≤2**  **no./total(%)** | **mRS≥3**  **no./total(%)** | ***P* value** |
| --- | --- | --- | --- | --- |
| Time from onset to emergency room (hours) | |  |  |  |
|  | ≤2 | 106/202(52.5) | 99/205(47.8) | 0.48 |
|  | 42041 | 44/202(21.8) | 59/205(28.5) |  |
|  | 42179 | 18/202(8.9) | 18/205(8.7) |  |
|  | >24 | 34/202(16.8) | 31/207(15.0) |  |
| Use of emergency ambulance | | 36/209(17.2) | 62/216(28.7) | 0.005 |
| Level of hospital admitted | |  |  |  |
|  | 1 | 59/205(28.8) | 46/210(21.9) | 0.11 |
|  | 2 | 113/205(55.1) | 137/210(65.2) |  |
|  | 3 | 33/205(16.1) | 27/210(12.9) |  |
| Ischemic stroke | |  |  |  |
|  | Intravenous thrombolysis | 2/151(1.3) | 4/140(2.9) | 0.43^b^ |
|  | Antiplatelet therapy after discharge(Aspirin) | 58/151(38.4) | 49/140(35.0) | 0.55 |
|  | Antiplatelet Therapy after discharge(Clopidogrel） | 6/151 (4.0) | 1/140(0.7) | 0.15 |
| Hemorrhagic stroke | |  |  |  |
|  | Surgery | 11(32.4) | 15(26.8) | 0.57 |
| Rehabilitation | | 74/209(35.4) | 113/216(52.3) | <0.001 |
| Duration of rehabilitation(weeks) | |  |  |  |
|  | <4 | 38/69(55.1) | 56/105(53.3) | 0.50^b^ |
|  | 4-8 | 7/69(10.1) | 17/105(16.2) |  |
|  | 8-12 | 1/69(1.4) | 4/105(3.8) |  |
|  | >12 | 23/69(33.3) | 28/105(26.7) |  |
| Inhospitalization in the previous year | | 48/209(23.0) | 58/216(26.9) | 0.36 |
| Outpatient attendance in the previous month | |  |  |  |
|  | Level 1 hospital | 49/209(23.4) | 39/216(18.1) | 0.17 |
|  | Level 2-3 hospital | 18/209(8.6) | 12/216(5.6) | 0.22 |

Abbreviation: CT indicates Computed Tomography, MRI indicates Magnetic Resonance Imaging, no. indicates number, RMB indicates Chinese Yuan Renminbi (exchange rate: US $1 equals to RMB6.4). *P* values are based on χ^2^ test if not indicated otherwise.

^a^*P* value based on unpaired *t* test

^b^*P* value based on Fisher’s exact test.

^c^*P* value based on Mann–Whitney *U* test.

^d^In multiple comparison, patients with one previous stroke had a higher proportion of poor functional outcome compared with first-ever strokes (*P*=0.008); patients with two or more previous strokes had a high probability of poor functional outcome compared with first-ever strokes (*P*=0.001); no significant difference was found between those with one previous stroke and with two or more previous stroke.
